# Supplementary material for: Antibiotic Prescriptions among China Ambulatory Care Visits of Pregnant Women: A Nationwide Cross-Sectional Study
Source: Antibiotics (Basel). 2021 May 19;10(5):601. doi: 10.3390/antibiotics10050601 (PMC8158752; doi:10.3390/antibiotics10050601)
Supplement: Supplementary file 1 [file antibiotics-10-00601-s001.zip › antibiotics-1218846-SI.pdf]

Appendix Table of Contents

Table S1. Number of included and excluded hospitals in different subgroups.

Table S2. Outpatient visits in included and excluded hospitals for various subgroups.

Table S3. Antibiotics prescribed for pregnant women and the categories.

Table S4. Percentages of Antibiotic prescriptions during pregnancy by age group and diagnosis.

Figure S1. Use of broad-spectrum antibiotics for different diagnosis categories.

Table S5. Prescription proportions of all antibiotic agents used by pregnant women

Appendix: Comparisons between the included and excluded hospitals in this study

Direct comparison of outpatient visits of pregnant women between the included and excluded hospitals was infeasible due to lack of diagnosis information in the excluded hospitals. Table S1 shows the numbers of included and excluded hospitals in different subgroups. Table S2 gives the outpatient visits in included and excluded hospitals for various subgroups. We used standardized mean difference, which represents small difference if the value is less than 0.2 [1], to indicate the significance of the difference between included and excluded hospitals. The results in the Table S1 and Table S2 indicated that there were no significant differences between the included and excluded hospitals except that more outpatient visits from the Central region were excluded due to lack of diagnosis information.

Table S1. Number of included and excluded hospitals in different subgroups

Subgroups	Hospitals included		Hospitals excluded		Standardized mean difference
	No. of hospitals	Proportion of hospitals (%)	No. of hospitals	Proportion of hospitals (%)	
Overall	128	100.0	66	100.0	—
Hospital level					0.1428
2	38	29.7	24	36.4	
3	90	70.3	42	63.6	
Regions of China					0.0902
Eastern	53	41.4	25	37.9	
Central	18	14.1	11	16.7	
Western	41	32.0	22	33.3	
Northeastern	16	12.5	8	12.1	

Table S2. Outpatient visits in included and excluded hospitals for various subgroups

Subgroups	All visits, n (%)[a]			Visits with antibiotics, n(%)[b]		
	Hospitals included	Hospitals excluded	Standardized mean difference	Hospitals included	Hospitals excluded	Standardized mean difference
Overall	168493940 (100.0)	70117632 (100.0)		18479758 (11.0)	7127483 (10.2)	0.0261
Age group			0.1161			
0-5	7937454 (4.7)	3502089 (5.0)		1957015 (24.7)	775446 (22.1)	0.0594
6-17	7911049 (4.7)	3462146 (4.9)		1298530 (16.4)	513705 (14.8)	0.0434
18-44	73867993 (43.8)	33986228 (48.5)		8804288 (11.9)	3707749 (10.9)	0.0317
45-64	51505516 (30.6)	19390844 (27.7)		4167463 (8.1)	1488537 (7.7)	0.0154
≥65	26594201 (15.8)	9690694 (13.8)		2194782 (8.3)	632176 (6.5)	0.0661
Unknown	677727 (0.4)	85631 (0.1)		57680 (8.5)	9870 (11.5)	0.1006
Gender of patients			0.0177			
Female	87664917 (52.0)	35868609 (51.2)		8307515 (9.5)	3225126 (9.0)	0.0168
Male	80671932 (47.9)	34176480 (48.7)		10151864 (12.6)	3898969 (11.4)	0.0362
Unknown	157091 (0.1)	72543 (0.1)		20379 (13.0)	3388 (4.7)	0.2959
Type of patients			0.0149			
Emergency	13478129 (8.0)	5327936 (7.6)		4042328 (30.0)	1278337 (24.0)	0.1354
Outpatient	155015811 (92.0)	64789696 (92.4)		14437430 (9.3)	5849146 (9.0)	0.0099
Hospital level			0.0262			
Secondary	13218795 (7.8)	6005674 (8.6)		1679655 (12.7)	735531 (12.2)	0.0139
Tertiary	155275145 (92.2)	64111958 (91.4)		16800103 (10.8)	6391952 (10.0)	0.0278
Regions of China			0.4592			
Eastern	94717757 (56.2)	36856158 (52.6)		11761093 (12.4)	4166081 (11.3)	0.0344
Central	9149698 (5.4)	12961475 (18.5)		1039203 (11.4)	1492795 (11.5)	0.0050
Western	48431349 (28.7)	17641749 (25.2)		4323362 (8.9)	1296869 (7.4)	0.0576

Northeastern	16195136 (9.6)	2658250 (3.8)		1356100 (8.4)	171738 (6.5)	0.0730
Year of visit			0.0455			
2014	11547018 (6.9)	5243222 (7.5)		1486769 (12.9)	605392 (11.5)	0.0406
2015	48417915 (28.7)	21113692 (30.1)		5518078 (11.4)	2213170 (10.5)	0.0293
2016	48077338 (28.5)	19813112 (28.3)		5170057 (10.8)	1970710 (9.9)	0.0265
2017	46878388 (27.8)	18556863 (26.5)		4851011 (10.3)	1793869 (9.7)	0.0227
2018	13573281 (8.1)	5390743 (7.7)		1453843 (10.7)	544342 (10.1)	0.0201

[a] Percentages in the parentheses are the proportions of outpatient visits within each subgroup.

[b] Percentages in the parentheses are the percentages of outpatient visits ended with antibiotic prescriptions.

Table S3. Antibiotics prescribed for pregnant women and the categories

Antibiotic categories	Antibiotics
**FDA Pregnancy Category B**	
Beta-lactam antibacterials, penicillins	Amoxicillin, Amoxicillin-clavulanate, Amoxicillin-dicloxacillin, Amoxicillin-flucloxacillin, Amoxicillin-sulbactam, Ampicillin, Ampicillin-probenecid, Azlocillin, Benzathine benzylpenicillin, Benzylpenicillin, Flucloxacillin, Lenampicillin, Mezlocillin, Mezlocillin-sulbactam, Phenoxymethylpenicillin, Piperacillin, Piperacillin-sulbactam, Piperacillin-tazobactam, Sulbenicillin, Sultamicillin, Ticarcillin-clavulanate
Monobactams and cephalosporins	Aztreonam, Cefaclor, Cefadroxil, Cefalexin, Cefalotin, Cefamandole, Cefathiamidine, Cefazedone, Cefazolin, Cefdinir, Cefepime, Cefetamet, Cefixime, Cefmenoxime, Cefmetazole, Cefminox, Cefodizime, Cefonicid, Cefoperazone, Cefoperazone-sulbactam, Cefoperazone-tazobactam, Cefotaxime, Cefotaxime-sulbactam, Cefotiam, Cefoxitin, Cefpiramide, Cefpodoxime, Cefprozil, Cefradine, Ceftazidime, Cefteram pivoxil, Ceftezole, Ceftizoxime, Ceftriaxone, Ceftriaxone-tazobactam, Cefuroxime, Latamoxef
Carbapenems	Meropenem
Macrolides and lincosamides	Azithromycin, Clindamycin, Dirithromycin, Erythromycin, Erythromycin cyclocarbonate, Erythromycin ethylsuccinate, Roxithromycin, Lincomycin
Imidazole derivatives	Metronidazole
Other antibiotics	Fosfomycin, Nitrofurantoin, Vancomycin
**FDA Pregnancy Category C**	
Carbapenems	Imipenem-cilastatin
Sulfonamides and trimethoprim	Sulfamethoxazole-trimethoprim
Macrolides and lincosamides	Clarithromycin
Quinolones	Antofloxacin, Ciprofloxacin, Enoxacin, Fleroxacin, Gatifloxacin, Levofloxacin, Moxifloxacin, Norfloxacin, Ofloxacin, Pazufloxacin, Pipemidic acid, Sparfloxacin
Imidazole derivatives	Tinidazole
Other antibiotics	Cefalexin-trimethoprim, Linezolid
**FDA Pregnancy Category D**	
Tetracyclines	Doxycycline, Minocycline, Tetracycline, Tigecycline
Aminoglycosides	Amikacin, Etimicin, Gentamicin, Isepamicin, Streptomycin
**Category N: not be assigned with any FDA Category**	
Macrolides	Kitasamycin
Carbapenems	Biapenem, Faropenem
Other antibiotics	Ornidazole, Levornidazole, Thiamphenicol

Table S4. Percentages of Antibiotic prescriptions during pregnancy by age group and diagnosis

	18-25 years		26-35 years		>35 years	
**Diagnosis categories***	**Outpatient visits (with antibiotics/all visits)**	**Antibiotic prescription rate in pregnancy, % (95% CI)**	**Outpatient visits (with antibiotics/all visits)**	**Antibiotic prescription rate in pregnancy, % (95% CI)**	**Outpatient visits (with antibiotics/all visits)**	**Antibiotic prescription rate in pregnancy, % (95% CI)**
**Tier 1 diagnoses that antibiotics are almost always indicated**						
Pneumonia	27/47	57.45 (42.18-71.74)	108/194	55.67 (48.38-62.78)	35/53	66.04 (51.73-78.48)
Urinary tract infections	435/1245	34.94 (32.29-37.66)	1031/3422	30.13 (28.59-31.70)	169/496	34.07 (29.91-38.43)
Certain bacterial diseases	882/1834	48.09 (45.78-50.41)	2034/4301	47.29 (45.79-48.80)	371/824	45.02 (41.59-48.49)
Other bacterial infections	3711/8572	43.29 (42.24-44.35)	4326/11523	37.54 (36.66-38.43)	1184/2931	40.40 (38.61-42.20)
All tier 1 diagnoses	5055/11698	43.21 (42.31-44.12)	7499/19440	38.58 (37.89-39.26)	1759/4304	40.87 (39.40-42.36)
**Tier 2 diagnoses that antibiotics are sometimes indicated**						
COPD	0/4	0.0 (0.0-60.24)	2/18	11.11 (1.38-34.71)	1/18	5.56 (0.14-27.29)
Acute sinusitis	10/21	47.62 (25.71-70.22)	72/183	39.34 (32.22-46.82)	17/58	29.31 (18.09-42.73)
Acute pharyngitis	104/271	38.38 (32.56-44.45)	565/1613	35.03 (32.70-37.41)	112/395	28.35 (23.96-33.08)
Acute otitis media	7/30	23.33 (9.93-42.28)	29/132	21.97 (15.23-30.00)	7/19	36.84 (16.29-61.64)
Other infectious diseases of the respiratory system	120/359	33.43 (28.56-38.57)	511/1582	32.30(30.00-34.67)	139/387	35.92 (31.13-40.92)
Infectious diseases of oral cavity and salivary glands	53/302	17.55 (13.43-22.32)	281/1446	19.43 (17.42-21.57)	40/218	18.35 (13.44-24.14)
Infectious gastroenteritis	20/183	10.93 (6.80-16.37)	77/567	13.58 (10.87-16.68)	16/76	21.05 (12.54-31.92)

Other infectious diseases of the digestive system	9/100	9.00 (4.20-16.40)	31/333	9.31 (6.41-12.95)	8/77	10.39 (4.59-19.45)
Acne	19/127	14.96 (9.25-22.37)	33/335	9.85 (6.88-13.56)	3/43	6.98 (1.46-19.06)
Impetigo	0/2	0.0 (0.0-84.19)	2/10	20.00 (2.52-55.61)	0/1	0.0 (0.0-97.50)
Other skin, cutaneous, and mucosal infections	14/135	10.37 (5.79-16.79)	104/832	12.50 (10.33-14.94)	26/147	17.69 (11.89-24.83)
Other infectious diseases that antibiotic may be indicated	1943/20660	9.4 0(9.01-9.81)	3312/67360	4.92 (4.75-5.08)	903/11121	8.12 (7.62-8.64)
All tier 2 diagnoses	2299/22194	10.36 (9.96-10.77)	5019/74411	6.74 (6.57-6.93)	1272/12560	10.13 (9.61-10.67)
**Tier 3 diagnoses that antibiotics are not indicated**						
Viral infections	10/1607	0.62 (0.30-1.14)	19/5406	0.35 (0.21-0.55)	4/625	0.64 (0.17-1.63)
Fungal infections	7/352	1.99 (0.80-4.05)	22/1118	1.97 (1.24-2.96)	6/184	3.26 (1.21-6.96)
Non-suppurative otitis media	2/10	20.00 (2.52-55.61)	15/69	21.74 (12.71-33.31)	4/12	33.33 (9.92-65.11)
Viral upper respiratory tract infection (URTI)	448/1427	31.39 (28.99-33.87)	1932/6284	30.74 (29.61-31.90)	428/1273	33.62 (31.03-36.29)
Influenza	0/2	0.0 (0.0-84.19)	0/7	0.0 (0.0-40.96)		
Acute bronchitis	92/231	39.83 (33.46-46.45)	723/1625	44.49 (42.06-46.95)	153/321	47.66 (42.09-53.28)
Allergy and asthma	15/253	5.93 (3.36-9.59)	58/1260	4.60 (3.51-5.91)	9/266	3.38(1.56-6.33)
Cough	12/133	9.02 (4.75-15.23)	138/916	15.07 (12.81-17.55)	29/191	15.18 (10.41-21.07)
Other non-infectious gastroenteritis	62/660	9.39 (7.28-11.88)	209/2106	9.92 (8.68-11.28)	33/373	8.85 (6.17-12.20)
Non-specific symptoms, signs of respiratory system[b]	7/126	5.56 (2.26-11.11)	25/388	6.44 (4.21-9.36)	9/78	11.54 (5.41-20.78)

Non-specific symptoms, signs of digestive system	83/1646	5.04 (4.04-6.21)	132/4017	3.29 (2.76-3.88)	32/546	5.86 (4.04-8.17)
Fever	108/362	29.83(25.16-34.84)	250/906	27.59 (24.70-30.63)	35/97	36.08 (26.58-46.46)
Procedures and surgeries not included elsewhere	123/1019	12.07 (10.13-14.23)	161/5993	2.69 (2.29-3.13)	60/1252	4.79 (3.68-6.13)
All other conditions not listed above	19057/815646	2.34 (2.30-2.37)	34359/3108290	1.11 (1.09-1.12)	10740/463277	2.32 (2.28-2.36)
All tier 3 diagnoses	20026/823474	2.43 (2.40-2.47)	38043/3138385	1.21 (1.20-1.22)	11542/468495	2.46 (2.42-2.51)
All conditions	27380/857366	3.19 (3.16-3.23)	50561/3232236	1.56 (1.55-1.58)	14573/485359	3.00 (2.95-3.05)

*Tier 1 diagnoses are conditions for which antibiotic is almost always indicated, such as pneumonia; tier 2 diagnoses are conditions for which antibiotic may be indicated, such as sinusitis; finally tier 3 diagnose are all other conditions for which antibiotic is almost never indicated.

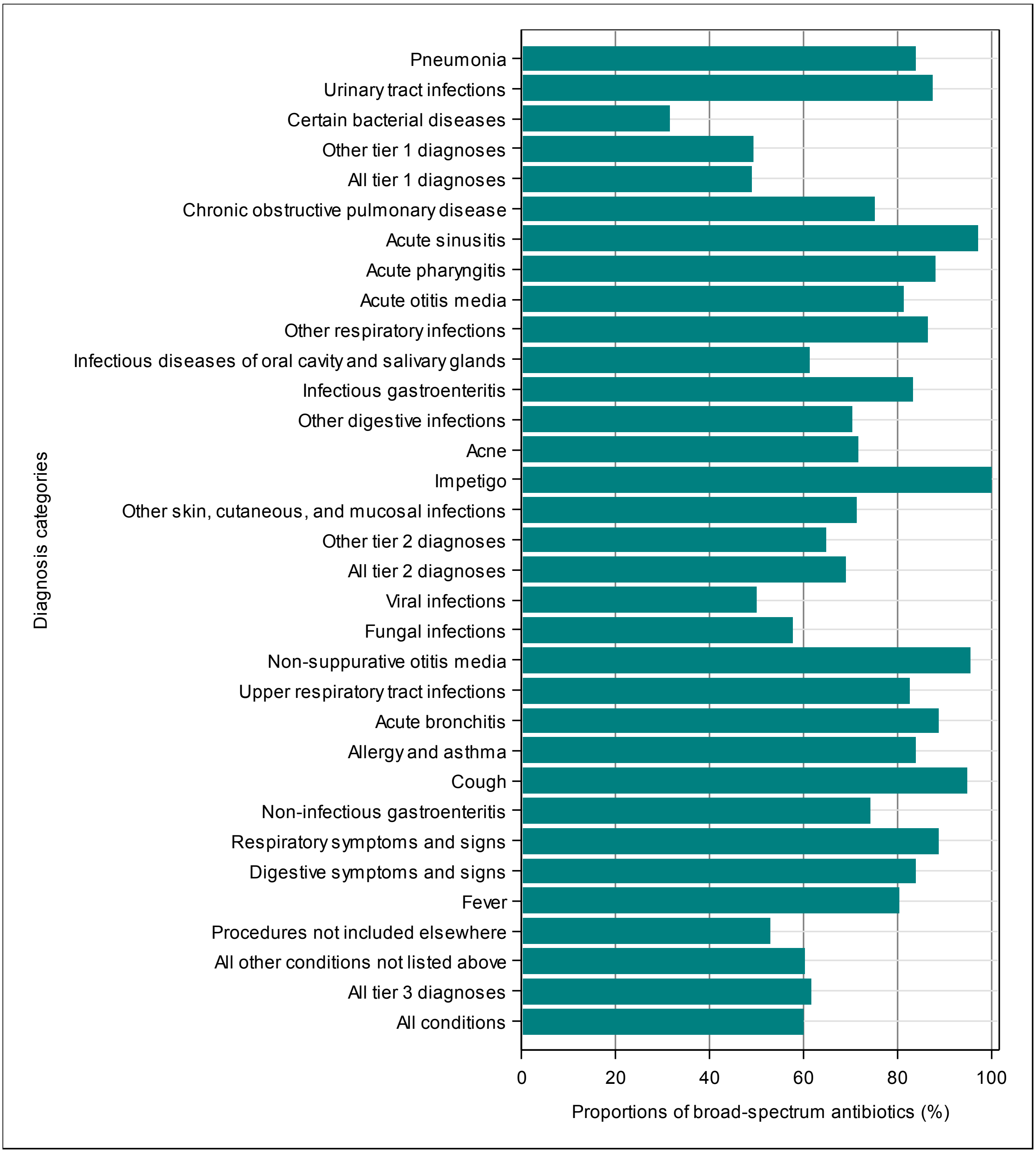

Figure S1. Use of broad-spectrum antibiotics for different diagnosis categories

Table S5. Prescription proportions of all antibiotic agents used by pregnant women

Antibiotics	Prescription frequency	Proportion of prescribing within categories, %	Proportion of prescribing among all antibiotics, %
**FDA Pregnancy Category B**			
Azithromycin	11055	14.05	8.48
Cefixime	10267	13.05	7.88
Cefdinir	10140	12.89	7.78
Metronidazole	9202	11.69	7.06
Cefuroxime	5868	7.46	4.50
Cefaclor	3371	4.28	2.59
Amoxicillin	3273	4.16	2.51
Clindamycin	2968	3.77	2.28
Benzylpenicillin	2716	3.45	2.08
Cefprozil	2141	2.72	1.64
Cefamandole	1687	2.14	1.29
Amoxicillin-clavulanate	1609	2.04	1.23
Benzathine benzylpenicillin	1535	1.95	1.18
Cefmetazole	1192	1.51	0.91
Cefradine	1137	1.44	0.87
Erythromycin	981	1.25	0.75
Aztreonam	953	1.21	0.73
Ceftriaxone	942	1.2	0.72
Cefalexin	848	1.08	0.65
Fosfomycin	822	1.04	0.63
Sultamicillin	538	0.68	0.41
Roxithromycin	473	0.6	0.36
Cefazolin	414	0.53	0.32
Cefpodoxime	375	0.48	0.29
Dirithromycin	322	0.41	0.25
Cefoxitin	313	0.4	0.24
Ceftazidime	312	0.4	0.24
Sulbenicillin	284	0.36	0.22
Cefodizime	269	0.34	0.21
Cefoperazone	245	0.31	0.19
Cefathiamidine	211	0.27	0.16
Amoxicillin-sulbactam	202	0.26	0.16

Cefotiam	194	0.25	0.15
Cefoperazone-sulbactam	185	0.24	0.14
Amoxicillin-flucloxacillin	157	0.2	0.12
Lincomycin	146	0.19	0.11
Cefotaxime	140	0.18	0.11
Piperacillin-sulbactam	115	0.15	0.09
Phenoxymethylpenicillin	112	0.14	0.09
Cefminox	110	0.14	0.08
Cefadroxil	108	0.14	0.08
Cefepime	93	0.12	0.07
Ceftizoxime	87	0.11	0.07
Flucloxacillin	83	0.11	0.06
Cefoperazone-tazobactam	74	0.09	0.06
Ceftriaxone-tazobactam	44	0.06	0.03
Latamoxef	41	0.05	0.03
Piperacillin-tazobactam	35	0.04	0.03
Mezlocillin-sulbactam	28	0.04	0.02
Meropenem	27	0.03	0.02
Cefotaxime-sulbactam	26	0.03	0.02
Cefalotin	26	0.03	0.02
Erythromycin ethylsuccinate	26	0.03	0.02
Cefteram pivoxil	22	0.03	0.02
Ceftezole	17	0.02	0.01
Cefmenoxime	16	0.02	0.01
Nitrofurantoin	14	0.02	0.01
Ampicillin-probenecid	13	0.02	0.01
Azlocillin	13	0.02	0.01
Piperacillin	12	0.02	0.01
Lenampicillin	10	0.01	0.01
Ampicillin	9	0.01	0.01
Cefpiramide	8	0.01	0.01
Mezlocillin	8	0.01	0.01
Cefonicid	7	0.01	0.01
Erythromycin cyclocarbonate	5	0.01	<0.01
Ticarcillin-clavulanate	5	0.01	<0.01
Cefetamet	4	0.01	<0.01
Vancomycin	4	0.01	<0.01
Amoxicillin-dicloxacillin	3	<0.01	<0.01

**FDA Pregnancy Category C**			
Levofloxacin	13068	50.02	10.03
Tinidazole	7796	29.84	5.98
Clarithromycin	1497	5.73	1.15
Enoxacin	1374	5.26	1.05
Ciprofloxacin	1264	4.84	0.97
Moxifloxacin	455	1.74	0.35
Ofloxacin	180	0.69	0.14
Pazufloxacin	159	0.61	0.12
Gatifloxacin	105	0.4	0.08
Norfloxacin	97	0.37	0.07
Fleroxacin	70	0.27	0.05
Imipenem-cilastatin	33	0.13	0.03
Antofloxacin	10	0.04	0.01
Sulfamethoxazole-trimethoprim	5	0.02	<0.01
Cefalexin-trimethoprim	4	0.02	<0.01
Pipemidic acid	4	0.02	<0.01
Linezolid	4	0.02	<0.01
Sparfloxacin	1	<0.01	<0.01
**FDA Pregnancy Category D**			
Etimicin	2911	81.91	2.23
Gentamicin	277	7.79	0.21
Minocycline	145	4.08	0.11
Doxycycline	127	3.57	0.10
Amikacin	63	1.77	0.05
Tetracycline	25	0.7	0.02
Isepamicin	4	0.11	<0.01
Tigecycline	1	0.03	<0.01
Streptomycin	1	0.03	<0.01
**Category N: not be assigned with any FDA Category**			
Ornidazole	21642	98.66	16.61
Levornidazole	134	0.61	0.10
Kitasamycin	120	0.55	0.09
Faropenem	29	0.13	0.02
Thiamphenicol	6	0.03	<0.01
Biapenem	5	0.02	<0.01

References

1. Yang D, Dalton J. A unified approach to measuring the effect size between two groups using SAS. SAS Global Forum 2012, 2012
